# Evaluating the efficacy of bioelectrical impedance analysis using machine learning models for the classification of goats exposed to Haemonchosis

**DOI:** 10.3389/fvets.2025.1584828

**Published:** 2025-05-30

**Authors:** Aftab Siddique, Phaneendra Batchu, Arshad Shaik, Priyanka Gurrapu, Tharun Tej Erukulla, Cornileus Ellington, Andrea L. Rubio Villa, Davia Brown, Ajit Mahapatra, Sudhanshu Panda, Eric Morgan, Jan Van Wyk, David Shapiro-Ilan, Govind Kannan, Thomas H. Terrill

**Affiliations:** ^1^Department of Agricultural Sciences, Fort Valley State University, State University Drive, Fort Valley, GA, United States; ^2^Institute for Environmental Spatial Analysis, University of North Georgia, Oakwood, GA, United States; ^3^Institute for Global Food Security, Queen’s University, Belfast, United Kingdom; ^4^Department of Veterinary Tropical Diseases, Faculty of Veterinary Science, University of Pretoria, Onderstepoort, South Africa; ^5^United States Department of Agriculture- Agriculture Research Services, Fruit and Tree Nut Research, Byron, GA, United States; ^6^Department of Poultry Sciences, Auburn University, Auburn, AL, United States

**Keywords:** bioelectrical impedance, machine learning, gastrointestinal parasites, *Haemonchus contortus*, veterinary diagnostics

## Abstract

Rapid identification and assessment of animal health are critical for livestock productivity, especially for small ruminants like goats, which are highly susceptible to blood-feeding gastrointestinal nematodes, such as *Haemonchus contortus*. This study aimed at establishing proof of concept for using bioelectrical impedance analysis (BIA) as a non-invasive diagnostic tool to classify animals at different levels of Haemonchosis. A cohort of 94 intact Spanish bucks (58 healthy; 36 Unhealthy; naturally infected with *H. contortus*) was selected to evaluate the efficacy of BIA through the measurement of resistance (Rs) and electrical reactance (Xc). Data were collected from live goats using the CQR 3.0 device over multiple time points. The study employed several machines learning models, including Support Vector Machines (SVM), Backpropagation Neural Networks (BPNN), k-Nearest Neighbors (K-NN), XGBoost, and Keras deep learning models to classify goats based on their bioelectrical properties. Among the classification models, SVM demonstrated the highest accuracy (95%) and F1-score (96%), while K-NN showed the lowest accuracy (90%). For regression tasks, BPNN outperformed other models, with a nearly perfect R^2^ value of 99.9% and a minimal Mean Squared Error (MSE) of 1.25e-04, followed by SVR with an R^2^ of 96.9%. The BIA data revealed significant differences in Rs and Xc between lightly and more heavily Unhealthy goats, with the latter exhibiting elevated resistance values, likely due to dehydration and tissue changes resulting from Haemonchosis. These findings highlight the potential of BIA combined with machine learning to develop a scalable, rapid, and non-invasive diagnostic tool for monitoring small ruminant health, particularly in detecting parasitic infections like *H. contortus*. This approach could improve herd management, reduce productivity losses, and enhance animal welfare.

## 1 Introduction

In warm and wet regions, small ruminants such as goats and sheep are especially vulnerable to parasitic infections, with *Haemonchus contortus* posing one of the greatest threats (1) globally. Commonly referred to as the barber pole worm due to its distinctive red and white striped appearance, *H. contortus* thrives in warm, humid environments, infecting the abomasum (the fourth stomach chamber) of ruminants (2, 3) by attaching to the stomach mucosa and feeding on the host’s blood, while secreting anticoagulant into the tissues, which results in substantial blood loss, through seeping of blood from each bite, for considerable periods of time. Over time, infected animals develop anemia, which severely impacts their overall health, productivity, and even survival (2). For farmers and livestock producers, infections from *H. contortus* and other gastrointestinal nematodes represent a significant economic burden, causing diminished growth rates, lowered reproductive success, reduced milk production, and death in the case of unsuccessful worm management.

Anemia is a hallmark sign of *H. contortus* infection in animals and is traditionally diagnosed using Hematocrit Analysis, a method that measures the packed cell volume (PCV) of blood (4, 5). The PCV analysis provides an estimate of the level of anemia, reflecting the degree to which the animal is suffering from blood loss. Although reliable, Hematocrit Analysis is labor-intensive, requiring invasive blood sampling and the expertise of trained personnel. This limits its scalability, especially for large herds, and introduces additional stress to the animals (6, 7). The cost and time involved in performing Hematocrit Analysis make it less practical for routine monitoring in large or resource-limited operations.

The more traditional approach to diagnosis of parasitic infections in small ruminants is the fecal egg count (FEC), which constitutes estimation of the number of parasite eggs present in an animal’s feces as an indication of the parasite burden within the host (8, 9). It is particularly useful for monitoring gastrointestinal nematodes, such as *H. contortus*, as the number of eggs passed in the feces correlates with the worm burden in the animal (10, 11). It is also useful as an indicator in farm monitoring programs, of the need for treatment and for monitoring the efficacy of deworming programs (12, 13).

However, like Hematocrit Analysis, FEC is labor-intensive, requiring specialized equipment in diagnostic laboratories and expertise to collect, process, and interpret fecal samples (14, 15). Additionally, FEC results can vary significantly depending on the animal’s diet, hydration status, and the time of day when samples are collected. Moreover, the accuracy of FEC is the reciprocal of the worm egg count, thus may decrease when the parasite burden is low, leading to potential underestimation of the effect low levels of infection (16).

To address the limitations of traditional diagnostic methods such as PCV and FEC, researchers have been exploring alternative technologies that offer rapid, non-invasive, and scalable solutions. One alternative comprises bioelectrical impedance devices for analysis (BIA), a technique that measures the electrical properties of biological tissues, specifically electrical resistance (R) and electrical reactance (Xc) (17, 18). BIA works by passing a small, painless electrical impulse through the body and measuring how the tissues oppose the flow of the current (19, 20). Measurements taken through BIA provide insights into the body’s composition, including water content, fat mass, and cellular health (21, 22), all of which can be affected by parasitic infections.

**Figure 1:**
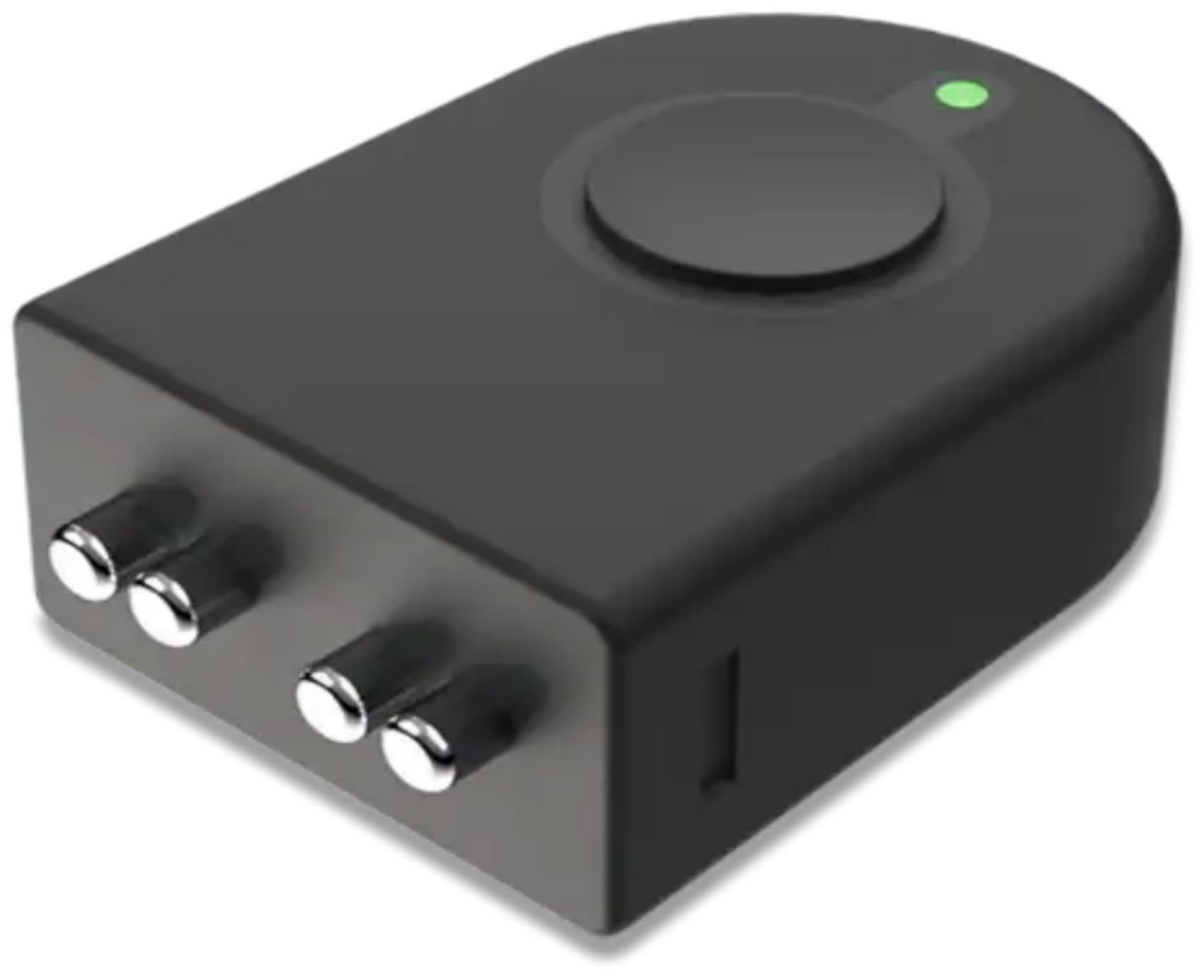
Bioelectrical impedance device.

In the case of *H. contortus*, the parasite’s blood-feeding behavior disrupts the host’s fluid balance and reduces the total volume of blood and numbers of red blood cells, which can alter the electrical properties of the host’s tissues. Thus, BIA may be a promising tool for detecting parasitic infections, as changes in the body’s fluid levels and tissue composition are directly related to the severity of the infection. Unlike Hematocrit Analysis or FEC, BIA can be performed quickly, without the need for invasive procedures or specialized personnel. This makes BIA particularly well-suited for large-scale herd management, where rapid, non-invasive diagnostics are crucial for maintaining animal health and productivity. The small BIA device (Figure 1) is held against the skin of the animal’s ear or tail for 1–2 s to rapidly take multiple the readings (5–10), with the R and Xc data automatically saved to the Cloud for analysis, which is completed by a commercial company. There is a cost for the device and the analysis (approximately $400 US), which should be substantially reduced as more users adopt the technology.

Given the advantages of BIA in terms of ease, speed, and non-invasiveness, this study as a proof of concept explored its potential in combination with machine learning (ML) techniques to classify goats as either Unhealthy or healthy. Machine learning algorithms, known for their ability to recognize complex patterns in large datasets, were applied to the bioelectrical impedance data collected from live goats. By training ML models on this data, the study aimed to develop an efficient, scalable, and accurate diagnostic tool for detecting parasitic infections in small ruminants. The implications of this research go beyond the detection of *H. contortus* infection. For instance, it seems possible that the successful application of BIA and ML could allow diagnosis of a wide range of health conditions in livestock, from other parasitic infections to metabolic disorders and nutritional deficiencies. By providing a rapid, non-invasive, and cost-effective diagnostic tool, this study lays the groundwork for the integration of advanced technologies in precision livestock farming, where early detection and proactive management are keys to maximizing productivity and animal welfare.

The following sections (1.1 to 1.5) provide the basic working principle for each of the different techniques used in the study.

### 1.1 Support vector machines (SVM)

Support Vector Machines (SVMs) are a category of supervised machine learning methods employed for classification, regression, and anomaly detection. The SVM technique is widely recognized in ML for its efficacy in handling both linear and nonlinear classification challenges. The fundamental concept of SVM is to identify a hyperplane in an N-dimensional space (where N denotes the number of features) that effectively segregates data points into various classes (23, 24). Winston (25) compares this approach to “Fitting the widest possible street,” thus elucidating the quadratic optimization challenge associated with hyperplane separation using the following Equation (1):


(1) wx+b=0

where w represents the weight vector, x is the input vector, and b signifies the bias factor (26). In SVM, hard-margin and soft-margin classifiers identify the optimal separation distances between classes, in this way to facilitate the classification of distinct patterns or features from an image (27).

Whereas numerous hyperplanes can delineate the data points of two classes in binary classification, more than two classes are considered in multi-class classification. The goal is to identify the hyperplane that maximizes the margin, defined as the maximum distance between the support vectors data points nearest to the hyperplane, without which the hyperplane’s position would be changed. Consequently, SVMs, the efficacy of which resides in their capacity to manage nonlinear data using the kernel trick for converting non-linearly separable data into a linearly separable format (28–30), are regarded as essential elements of the dataset.

### 1.2 K-nearest neighbor (K-NN)

K-Nearest Neighbors (KNN) is a basic and efficient non-parametric, supervised machine learning classifier which utilizes proximity to categorize or forecast the group to which a data item is allocated. The method operates by retaining all existing data points and categorizing new instances according to their similarity, generally assessed by distance metrics such as Euclidean, Manhattan, Minkowski, or Hamming distances (31, 32). The primary objective of KNN is to determine the nearest neighbors to a specified query point and subsequently allocate a class label. These distance functions facilitate the evaluation of proximate points, with the ultimate categorization established using a majority vote mechanism among the nearest neighbors. Each data point is categorized according to the predominant category among its closest neighbors, as established by the selected distance metric. The selection of K substantially influences prediction accuracy, lower values render the model susceptible to noise, whereas higher values elevate processing requirements. In datasets comprising two classes, researchers frequently select an odd value for K to prevent ties. Nonetheless, a limitation of KNN is that its processing performance may significantly diminish as the dataset size increases (22, 30, 32, 33).

### 1.3 Back propagation neural networks (BPNN)

Back Propagation Neural Networks (BPNNs) are the part of Artificial Neural Networks (ANNs) that employ the backpropagation method for training purposes. They are well acknowledged for their efficacy in deep learning models. A BPNN comprises a minimum of three layers of nodes: an input layer, one or more hidden layers, and an output layer. Every node, known as an artificial neuron or perceptron, is interconnected by weighted linkages, which are modified throughout the training process (28, 29, 34, 35). Multilayer Perceptrons employ the backpropagation method, consisting of two phases: a forward pass, in which input data is processed through the network to produce an output, and a backward pass, during which the error (the differences between the anticipated and actual output) flows backward through the network to adjust the weights. This modification is executed utilizing optimization techniques, including gradient descent. Backpropagation Neural Networks are advantageous due to their capacity to learn and represent complicated non-linear relationships in biological data in between values (34, 35). Upon completion of training, algorithms may generate precise predictions when presented with novel data, rendering them exceptionally adaptable and versatile for real life usage. For a comprehensive elucidation of BPNN functionality, refer to Siddique et al. (30). These models demonstrate proficiency in tasks necessitating pattern recognition and are trained utilizing features derived from diverse datasets, such images, numerical data, or text (36).

### 1.4 Extreme gradient boosting (XGBoost)

Extreme Gradient Boosting (XGBoost) is a complex delicate ML technique derived from the gradient boosting framework. The fundamental idea is to enhance forecast performance by incrementally constructing an ensemble of decision trees, with each tree rectifying the weaknesses of its predecessors. In the training process, XGBoost initially establishes a rudimentary model (often a constant value) and subsequently computes the residuals or discrepancies between the predicted and actual values. In succeeding steps, a new decision tree is fitted to minimize these residuals using gradient descent, a method that modifies the model by pursuing the direction of maximal error reduction. New trees are incorporated into the ensemble to forecast the residuals, while the model adjusts the weights of misclassified examples to mitigate subsequent errors (37).

Extreme Gradient Boosting integrates various distinctive optimizations. It employs regularization approaches (L1 and L2) to mitigate overfitting, hence assuring the model generalizes effectively to novel data (38). The algorithm adeptly manages absent values and sparse datasets, autonomously identifying the optimal trajectory through the decision tree in the absence of data. The XGBoost also executes tree pruning, ceasing tree growth when additional splits yield negligible enhancements, so improving both performance and efficiency (36). Extreme Gradient Boosting is esteemed for its scalability and quickness due to its capacity for parallel data processing, rendering it an optimal selection for managing extensive datasets and intricate issues. These qualities have rendered XGBoost highly esteemed in both academic research and industrial applications, particularly in regression and classification tasks (36, 37, 80).

### 1.5 Keras deep learning model

Keras, an advanced deep-learning framework, is designed to adapt to a wide range of problem domains, providing a streamlined approach to the development and training of ANNs. Built upon foundational libraries, like TensorFlow, it offers an intuitive interface for assembling, training, and deploying neural networks (38–40). A standard Keras model consists of several layers, including input, hidden, and output layers, which can be easily stacked to create complex architectures such as Convolutional Neural Networks (CNNs), Recurrent Neural Networks (RNNs), or Multilayer Perceptrons (MLPs) (38, 39). Keras models are trained via backpropagation, where errors are computed during a forward pass and transmitted backward through the network to modify weights via optimization methods, such as gradient descent. Its adaptability in integrating various activation functions, loss measures, and optimizers makes it suitable for a wide range of problems, such as image classification, text production, and numerical prediction (39, 40). The Keras model also facilitates the addition of pre-trained models, transfer learning, and custom layers, reassuring developers that it can be applied to their specific needs (41).

Keras, in contrast to Back Propagation Neural Networks (BPNNs), offers a more comprehensive and versatile framework for creating modern deep learning architectures. The BPNNs primarily emphasize the backpropagation technique and are generally linked to classic Multilayer Perceptrons (MLPs) (28, 30). They function with static designs and prioritize training by backpropagation (30). In contrast, Keras allows users to construct and explore other architectures, including CNNs and RNNs, which are superior for applications like image recognition and sequential data analysis (38). Keras simplifies numerous complexities associated with model training, including weight initialization, optimization techniques, and parallel calculations, which are often manual tasks in conventional BPNNs (39, 40). This versatility and comprehensive approach make Keras a more potent and versatile instrument for tackling modern deep-learning issues, instilling confidence in its capabilities among developers, data scientists, and machine-learning practitioners (38–40).

## 2 Materials and methods

### 2.1 Study design

Prior to the start of this study, all animal use protocols were approved by the Fort Valley State University (FVSU, Fort Valley, GA, USA) Agricultural and Laboratory Animal Care and Use Committee (ALACUC approval number WI-R-02-23).

**Figure 2:**
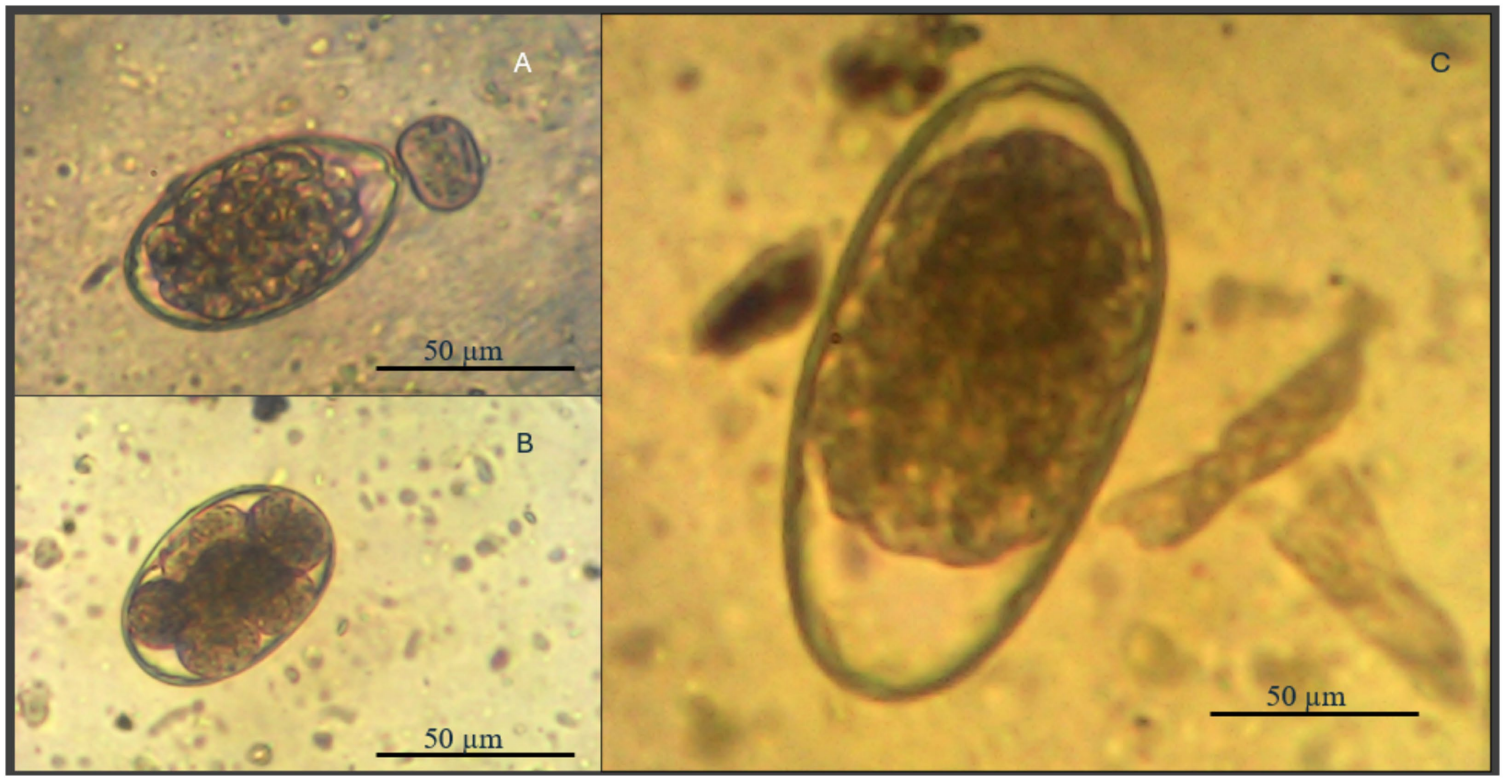
Composite image of gastrointestinal nematode egg types recovered from fecal samples of goats using the McMaster technique. **(A)** Haemonchus contortus; **(B)** Nematodirus and **(C)** Trichostrongylid spp. at 50 μm scale [images were referenced from Buchmann et al. (45)].

A total of 94 intact Spanish goat bucks (58 healthy; 36 Unhealthy) (24 months old; 36–50 kg) were allowed to graze the same grass pasture at the FVSU Agriculture Technology Center farm from September through December 2023. Prior to this period (April–August, 2023), the two sets of goats were maintained on separate pastures with one group (58, healthy) dewormed regularly using commercially available dewormers at prescribed doses (Brand Name: Cydectin®; active ingredient: Moxidectin 1 mg/mL (approved by FDA); given dose as recommended: 0.2 mg/Kg body weight), while the second group (36-unhealthy) was allowed to pick up a natural infection with blood and fecal samples collected weekly from individual animals and analyzed for packed cell volume (PCV) and fecal egg counts (FEC), respectively, to monitor parasitic infection levels (42–44). To confirm the morphological identity of strongyle-type eggs, selected samples were imaged under a compound microscope at 100x magnification using a camera mounted on the eyepiece (1,600x digital zoom). The size of the eggs was estimated by comparing them to a calibrated reference image containing a 50 μm scale bar. The observed eggs measured approximately 70–90 μm in length, with an elliptical shape, thin shell, and multi-blastomere structure—characteristics consistent with trichostrongylid nematodes. Based on morphology, regional prevalence, and clinical signs (anemia), the infections were presumptively attributed to *Haemonchus contortus* Eggs consistent with trichostrongylid morphology were observed (Figure 2), and based on size and morphology, they were presumptively identified as *Haemonchus contortus*, a parasite endemic to the region and commonly associated with anemia in small ruminants (45, 46). Goats in the second group were also evaluated weekly using the FAMACHA system, and only animals scored as FAMACHA 4 s and 5 s were dewormed. Once both treatment groups were combined to graze the same pasture (September–December), only the healthy goats’ parasite infection status was monitored, with FEC and PCV on individual animals determined monthly.

At the end of the combined grazing period (December 5, 6, and 11, 2023), bioelectrical impedance readings were collected on both healthy and unhealthy animals, and then machine learning (ML) models, including Support Vector Machines (SVM), Back Propagation Neural Networks (BPNNs), k-Nearest Neighbors (K-NN), Extreme Gradient Boosting (XGBoost), and Keras deep learning models were trained and evaluated to determine their ability to classify goats as healthy or unhealthy based on their bioelectrical properties, especially electrical resistance (Rs) and electrical reactance (Xc). A total of 1,540 observation points were collected from the bucks, consisting of 917 healthy goat observation points and 623 Unhealthy goat observation points.

### 2.2 Data collection and preprocessing

Each goat was subjected to multiple bioelectrical impedance analysis (BIA) measurements during the study period, with readings taken from either the ear or tail. As the study was conducted on the same animals over several weeks, a minimum of 10 readings per area (ear and tail) per animal was set to ensure measurement reliability. These readings were averaged for each animal to provide a consistent and representative value for analysis. Regarding the use of these measurements within the study, the dataset was divided into training and testing sets using a standard 80/20 split. The training set was used to train machine learning models, while the testing set was used to evaluate model performance. The nested cross-validation approach ensured that the models were optimized without overfitting, providing reliable performance metrics. Data collection was conducted using a BIA device and an online cloud-based server specifically designed by the BIA device provider (Certified Quality foods, Clinton Twp, MI, Figure 1). For classification and regression tasks, features were scaled using standard techniques, like normalization, to ensure uniformity across the dataset (47). The data was collected by placing the device against the skin of the underside of each goat’s ear and tail, where the body hairs are minimum, to ensure the electrical conductance.

For instance, BIA has been successfully used in pigs (81), rabbits (83), and dogs (84), where similar anatomical sites were chosen to enhance signal reliability and measurement precision. BIA has been widely applied to various animal species for diverse purposes. In pigs, it has been used to evaluate carcass composition, including fat and lean mass prediction (81). In rabbits, BIA has been utilized to monitor body condition and hydration status (Kopp et al., 2021). For dogs, BIA has been employed to assess body composition in veterinary practice, providing a non-invasive alternative to traditional body condition scoring (Ward et al., 2020). BIA has also been applied in fish to monitor body composition without sacrificing the animals (82). This versatility underscores BIA’s potential as a diagnostic tool across species. This step was critical, especially for ML algorithms like SVM and KNN, which are sensitive to feature scaling (48, 49). The target variable ‘y-test’ was adjusted, based, as follows, on the problem type: while classification models predicted categorical labels, regression models predicted continuous values (50). A 10-fold nested cross-validation approach was employed to reduce overfitting and evaluate model performance comprehensively. The latter method included an inner loop for hyperparameter optimization and an outer loop for model evaluation, thus providing more reliable performance estimates (51, 52).

The statistical analyses were also conducted with PROC GLIMMIX in SAS (Version 9.4; SAS Institute Inc.) to evaluate the impact of the animal condition (healthy versus unhealthy) on Rs and Xc with a set significance level of *P* equal to 0.05. Furthermore, the Poisson distribution with a logarithmic link function was employed to describe the response variables, based on the characteristics of the data; least squares mean (LS Means) were computed for each condition; Tukey–Kramer corrections were applied to address multiple comparisons. Furthermore, PROC MEANS was employed to compute descriptive statistics, encompassing the mean, standard deviation, and standard error for each variable.

### 2.3 Model development and pipeline

The model development process comprised two primary components, namely, classification and regression tasks, examined by several ML methods.

#### 2.3.1 Classification models pipeline

The Backpropagation Neural Networks, SVM, and KNN models used for classification-based analysis, were each evaluated using accuracy and AUC-ROC (Area Under the Receiver Operating Characteristic Curve) scores (53). The ‘classification_summary’ function executed the process of predicting the test set outcomes and calculated the respective performance metrics (54). The accuracy was computed using the ‘accuracy_score’ function from the scikit-learn library (54, 55), which provides a direct comparison between the predicted and actual labels (52). For models capable of generating probability estimates (e.g., SVM and BPNN), the AUC was calculated using the ‘roc_auc_score’ function, providing insight into how well the model distinguishes between different classes (56, 57). For models without probabilistic outputs (like KNN), the AUC score was marked as ‘N/A’ (50). The results for each model were compiled into a ‘Panda DataFrame’ to allow for the comparison of classification accuracy and AUC values (58).

#### 2.3.2 Regression models pipeline

The regression pipeline incorporated modified models for regression-based analysis, including BPNN, SVM, KNN, XGBoost, and a Keras-based Neural Network (37, 59). Furthermore, the ‘regression_summary’ function evaluated these models using two primary performance metrics: the R^2^ score and Mean Squared Error (MSE), of which the former score measured the proportion of variance in the test data explained by the model (60), and MSE quantified the average squared differences between the predicted and actual values, thus highlighting the overall prediction error (61). This pipeline facilitated the comparison of model performance by storing these results in a ‘Pandas DataFrame’ and providing a clear view of the most effective regression algorithms for the given dataset (58). The integration of a Keras Neural Network is particularly useful in exploring the capabilities of deep learning approaches in solving regression tasks (62).

#### 2.3.3 Nested cross-validation

A 10-fold nested cross-validation method was employed to enhance model resilience and mitigate the possibility of overfitting (51, 63). In the outer loop thereof, the dataset comprising 1,540 observation points was partitioned into 10 equal folds, each of which was utilized once as the test group, while the remaining nine folds or groups were employed for training. Hyperparameter optimization for the inner loop was conducted using an additional 10-fold cross-validation within the training set, in this way to ensure that the model’s parameters were refined without incorporating test data into the training phase (51). By considering both model selection and model evaluation, this nested technique yielded more dependable performance estimates (50, 51).

The layered cross-validation technique facilitated thorough optimization of model hyperparameters, especially for models like SVM and XGBoost, which necessitate meticulous parameter selection for peak performance (26, 37). The inner cross-validation loop guaranteed that, by model selection on optimal parameters derived from training data, the performance assessment in the outer loop (63) was not affected. This validation method was essential for both classification and regression processes to guarantee that the final performance measured appropriately represented the model’s generalization capacity (52). The performance measures from all outer folds were summed to yield a reliable assessment of the model’s efficacy throughout the complete dataset (51). This complex validation method produced more dependable models, especially in instances where the dataset used demonstrated variability (64). By also improving the confidence in model predictions for both classification and regression tasks, it was rendered a significant element of our investigation (52, 59, 64).

## 3 Results and discussion

Prior to the bioelectrical impedance analysis (BIA) in this study, healthy goats exhibited a significantly lower fecal egg count (FEC) of 40.83 eggs per gram (epg) compared to unhealthy (more Unhealthy) goats, which presented an FEC of 2917.65 epg (*F* = 41.07, *p* < 0.001). In addition, healthy goats had a blood packed cell volume of 23.80%, while the unhealthy group had an average PCV of 20.09% (*F* = 19.31, *p* < 0.001). As these procedures are considered the gold standard for determining level of parasitic infection, particularly for *H. contortus*, they were used to validate the machine learning (ML) models with the BIA data.

The PROC GLIMMIX method revealed a significant impact of condition (healthy vs. unhealthy) on electrical resistance (Rs) (*F* = 635.36, *p* < 0.0001). The latter goats demonstrated a significantly higher least squares mean electrical resistance (5.5321 ± 0.002520 SE) in comparison to healthy ones (5.4483 ± 0.002166 SE). The difference between the two categories was significantly different (Tukey-adjusted *p* < 0.0001), with the less healthy goats exhibiting an increase of 0.08377 units in electrical resistance. The PROC MEANS approach yielded descriptive statistics, revealing that, while the mean Rs for healthy goats was 232.37 ± 5.77 SE, that of the unhealthy goats had an average mean Rs of 252.68 ± 7.31 SE, hence corroborating the enhanced electrical resistance in animals with a greater parasitic infection.

This study results thereof indicate that level of parasitic infection markedly affects the bioelectrical impedance parameters of goats, especially that of electrical resistance (Rs). The significant elevation in Rs of the more heavily Unhealthy, relative to healthy goats, may potentially be attributable to blood loss, dehydration, and tissue changes induced by the gastrointestinal nematode parasite infection (65). By feeding on the host’s blood, anemia and lower fluid volume result, hence elevating tissue electrical resistance when electrical current encounters increased opposition in drier, less hydrated tissues (66, 67, 85).

A notable difference in electrical reactance (Xc) was also observed between healthy and unhealthy goats (*F* = 11.12, *p* = 0.0009), in that the latter had greater least squares mean electrical reactance (3.9628 ± 0.005524 SE) compared with the healthy animals (3.9388 ± 0.004608 SE), although the disparity was less pronounced than that observed for electrical resistance. In addition, the Tukey-adjusted comparison indicated statistical significance (*p* = 0.0009), with a mean difference of 0.02399 units in Xc. Descriptive statistics using PROC MEANS indicated mean Xc values of 51.36 ± 4.26 SE for healthy goats and 52.60 ± 5.23 SE for unhealthy goats, the rise of which in Xc in goats with a greater parasitic infection was statistically significant, although the impact was lower compared to that of Rs (Table 1). Electrical reactance indicates cell membrane integrity and fluid distribution, and the slight rise in Xc implies that parasite infections exert a limited influence on the capacitive characteristics of tissues (67). This may result from tissue injury and cell membrane disruption, which influence the storage of electrical current in the tissues, however the impact is less significant than electrical resistance (67–71).

**Table 1:** Comparison of bioelectrical impedance parameters (Electrical resistance and Electrical reactance) between healthy and unhealthy (more heavily Unhealthy) goats, measured using bioelectrical impedance analysis (BIA).

Parameter	Condition	
	Healthy	Unhealthy
Electrical resistance (Rs)	232.37 ± 5.07^b^	252.67 ± 7.32^a^
Electrical reactance (Xc)	51.36 ± 4.26^y^	52.60 ± 5.24^x^

Superscript on mean value for condition is significantly different (*p* < 0.009) for electrical resistance and electrical reactance between healthy and unhealthy (heavily unhealthy) goats.

The results of bioelectrical impedance analysis (BIA) highlight the influence of gastrointestinal nematodes on the health of small ruminants, as indicated by markedly increased fecal egg counts (FEC) and diminished packed cell volume (PCV) in infected goats. Although these characteristics are not pathognomonic, suggesting they are not solely indicative of gastrointestinal nematode infestations, they are broadly acknowledged as dependable diagnostic markers in parasitology. This investigation utilized FEC and PCV as the principal diagnostic tools to verify the presence of parasites, aligning with their recognized status as the gold standard for evaluating gastrointestinal nematode infections, especially in areas where *Haemonchus contortus* are prevalent.

The significant differences in FEC and PCV between healthy and unhealthy groups further validate their effectiveness in assessing parasite burden and associated anemia. The results underscore the diagnostic potential of BIA as a non-invasive, supplementary tool for recognizing the physiological effects of parasitism. This method offers a rapid and scalable alternative to traditional diagnostic methods.

Moreover, the integration of BIA with machine learning models in this study significantly enhances the diagnostic potential, enabling precise classification of goats based on their bioelectrical traits. This combination presents a robust, non-invasive approach for health monitoring in small ruminants, capable of swiftly identifying the impacts of parasite diseases on animal health.

### 3.1 Performance evaluation matrices

Table 2 summarizes the classification performance of the models, presenting the Accuracy, Precision, Recall, and F1-Score for each model. Among the models, the SVM exhibited superior performance, achieving an accuracy of 95%, a precision of 93%, and an F1-Score of 96%, demonstrating its robust capacity to differentiate between healthy and Unhealthy goats. XGBoost achieved an accuracy of 94% and an F1-Score of 95%, indicating the efficacy of ensemble approaches in managing intricate datasets (37, 59). The BPNN model demonstrated a high level of performance, with an accuracy of 92% and an F1-Score of 94%, positioning it as a competitive alternative for classification tasks (72, 73). Keras DL attained an accuracy of 91% and an F1-Score of 93%, indicating that deep learning models can perform effectively, although they were unable to exceed SVM or XGBoost (74, 75). Ultimately, K-NN attained the lowest accuracy of 90%, suggesting it underperformed relative to more advanced models such as SVM and XGBoost, perhaps because to its simplicity and susceptibility to data noise and non-linearity in data (74).

**Table 2:** Comparison of different classification performance matrices for classification of goat health condition (healthy vs Unhealthy).

Models	Accuracy (%)	Precision (%)	Recall (%)	F1-Score (%)
SVM	95	93	94	96
BPNN	92	91	92	94
K-NN	90	88	89	92
XGBoost	94	92	93	95
Keras DL	91	90	91	93

Table presents the results for the different model performance matrices for classifying goat health to detect Unhealthy vs healthy goats. Accuracy, Precision, Recall, and F1 Score are performance metrics represented in percentage that assess the model’s classification efficiency. The F1 score is the harmonic mean of Precision and Recall, providing a balanced measure of the model’s ability to classify data correctly.

The robust efficacy of SVM and XGBoost in classification tasks can be linked to several factors. Support Vector Machine (SVM) operates by identifying the ideal hyperplane that maximizes the margin between classes, rendering it particularly successful for linearly separable datasets (26, 50, 59, 76). Considering that bioelectrical impedance measurements probably demonstrate unique patterns between healthy and Unhealthy goats, the capability of SVM to establish a definitive separation in the feature space enables it to attain high accuracy. Furthermore, SVM excels with high-dimensional data, potentially demonstrating its higher efficacy compared to simpler models such as K-NN (33, 50).

XGBoost demonstrated excellent performance, which is anticipated due to its capacity to manage intricate, non-linear interactions via boosting. XGBoost captures complex patterns in the dataset by systematically rectifying errors from prior iterations (37, 58). This is especially advantageous in biological datasets because nuanced variations in characteristics can significantly influence classification (50, 77). The BPNN and Keras deep learning models exhibited marginally reduced performance compared to SVM and XGBoost, although they still demonstrated competitive outcomes (73). Neural networks probably encapsulate intricate, non-linear relationships within the data; yet their efficacy may be affected by the selection of hyperparameters or the dimensions of the neural network. The K-NN method, due to its reliance on proximity-based judgments, had difficulties managing the dataset’s complexity, maybe accounting for its lower accuracy relative to other models (60, 75, 80).

Table 3 shows the efficacy of the models in forecasting the extent of parasitism, as indicated by the R-squared (R^2^) value and Mean Squared Error (MSE). The BPNN model demonstrated superior performance, achieving a R^2^ value of 99.9% and a minimal MSE of 1.25e-04, signifying its exceptional predictive accuracy about the health status of goats (73). SVR demonstrated strong performance, achieving a R^2^ value of 96.9% and a minimal MSE of 7.69e-03, indicating its high reliability as a regression model (37, 59). XGBoost and Keras DL attained R^2^ values of 89.2 and 88%, respectively, with moderate MSE values, suggesting that although these models exhibited better performance, they lacked the precision of BPNN or SVR. K-NN regression exhibited the poorest performance, with a R^2^ value of 83% and a higher MSE of 3.30e-02, indicating its worse ability to describe the intricate correlations between bioelectrical data and goat health problems relative to the other models (33).

**Table 3:** Comparison of different regression model performance matrices for classification of goat health condition (Healthy vs Unhealthy).

Models	R square value	MSE
SVR	96.9	7.69e-03
BPNN	99.9	1.25e-04
KNN	83.0	3.30e-02
XGBoost	89.2	0.025
Keras DL	88	0.027

The table presents a comparison of various models used for regression analysis, with their corresponding R-squared values and Mean Squared Error (MSE). These results provide insights into the performance of different algorithms, highlighting the superior accuracy of the BPNN model in this specific context.

The BPNN regression model was the most successful model in regression tasks, obtaining a nearly perfect R^2^ value of 99.9%. This suggests that the neural network model was able to accurately anticipate the degree of parasitism by capturing nearly all the variance in the dataset (73, 75). The success of BPNN in regression can be attributed to its capacity to learn complex, non-linear patterns, which is particularly advantageous in biological data that involve interactions between various physiological parameters. BPNN outperformed other models in regression tasks because neural networks are well-suited for encoding these intricate relationships (78).

Additionally, SVR demonstrated better performance, attaining a R^2^ value of 96.9%. SVR operates similarly to SVM, but it is designed to identify a function that deviates from the true data points by a small margin for continuous data (78). The efficacy of SVR in predicting parasitism severity may have been influenced by its capacity to manage outliers and noisy data. XGBoost and Keras DL also demonstrated strong predictive potential; however, they were unable to achieve the same level of precision as BPNN or SVR. This could be attributed to the hyperparameter tuning limitations or the complexity of the data (37, 60). However, K-NN regression encountered difficulty with this task, most likely due to its dependence on local averaging, which may not adequately capture the global trends apparent in the dataset, as opposed to more advanced models such as BPNN and SVR (31, 74).

## 4 Conclusion

Bio-electrical impedance analysis (BIA) offers a promising approach to transforming parasitism detection and livestock management by providing a rapid, scalable, and non-invasive diagnostic technology. While traditional methods such as fecal egg count (FEC) and hematocrit analysis are recognized as gold-standard techniques, they require substantial time, expertise, and laboratory facilities, making them less practical for large-scale or resource-limited settings. BIA, when combined with machine learning, provides real-time diagnostic insights that enhance decision-making. However, it is important to emphasize that BIA is most effective when used alongside conventional diagnostic methods, such as FEC, which remain essential for accurate etiological identification of parasite species. This integrated approach enables farmers and veterinarians to monitor herd health more efficiently, promptly identify and treat parasitic infections, minimize production losses, and improve animal welfare.

This work highlights the diagnostic capability of BIA in detecting goat parasitism through identification of notable physiological alterations linked to parasite infections. Machine learning algorithms accurately categorized goats as healthy or Unhealthy using bioelectrical impedance metrics, with Support Vector Machines (SVM) and Backpropagation Neural Networks (BPNN) demonstrating the highest level of precision. The BPNN attained nearly flawless diagnostic accuracy, with an R^2^ value of 99.9%, illustrating its capacity to model the intricate, non-linear relationships between bioelectrical characteristics and parasitism. These findings underscore BIA’s efficacy as a dependable diagnostic instrument for evaluating the physiological effects of parasitism.

Parasitic infections modify the electrical properties of tissues, as demonstrated by notable disparities in electrical resistance (Rs) and reactance (Xc) between healthy and Unhealthy goats. The elevated Rs in Unhealthy goats indicate dehydration and blood loss due to infection with gastrointestinal nematodes such as *Haemonchus contortus*, leading to anemia and diminished fluid retention in tissues. The capability of BIA to identify these alterations renders it an effective instrument for non-invasive, field-ready diagnostics, especially when integrated with advanced machine learning algorithms such as SVM and XGBoost, which are proficient in analyzing high-dimensional datasets and discerning subtle variations in bioelectrical measurements (79).

This study highlights the effectiveness of BIA in diagnosing parasitism through changes in tissue electrical properties. However, it is important to acknowledge that hydration levels in goats can be influenced by factors beyond parasitism, such as ambient temperature, nutritional intake, lactation status, or preexisting health conditions. These factors may introduce variability in BIA measurements, which must be addressed when interpreting results. The urgency and significance of addressing this variability in BIA measurements is clear, and future studies are needed to develop corrective models or standardized methods to enhance the reliability of BIA as a diagnostic tool.

Use of BIA should potentially provide various practical benefits to livestock producers, especially for extensive herd management. Its non-invasive characteristics obviate the necessity for blood sampling or fecal collection, hence diminishing animal stress and the likelihood of handling-related accidents. The expedited data collection procedure renders BIA scalable and efficient for health monitoring in extensive herds. Integration with machine learning guarantees elevated precision and impartiality in diagnosis, reducing variability linked to manual diagnostic methods. Moreover, BIA diminishes reliance on skilled workers and laboratory facilities, rendering it particularly appropriate for resource-constrained environments. Although BIA demonstrates considerable potential, it is not without restrictions. It offers an indirect evaluation of parasitism, indicating physiological alterations instead of pinpointing specific parasites. Complementary diagnostic procedures such as fecal egg count (FEC) or pathogen-specific assays may still be necessary for accurate etiological identification. Environmental influences, such as fluctuations in hydration levels, may affect BIA measurements and must be considered in practical applications. Moreover, although BIA devices necessitate an initial expenditure, the long-term financial benefits derived from decreased labor and enhanced herd health can counterbalance this expense.

Despite these challenges, BIA is viable for agricultural use owing to its mobility, user-friendliness, and integration with contemporary farm management systems. The method’s scalability ensures its sustainability for prolonged application in herds of diverse sizes. It facilitates early diagnosis of parasitism in individual animals, diminishing the necessity for routine blanket treatments, promoting sustainable parasite management methods, and supporting worldwide initiatives to mitigate anthelmintic resistance of gastrointestinal nematodes. This work highlights the diagnostic capability of BIA as a swift, non-invasive, and scalable instrument for detecting parasitism in goats. Its incorporation of machine learning improves precision and dependability, rendering it an effective solution for contemporary livestock management. Future endeavors will enhance the technology, mitigate limits, and investigate its wider applicability to additional species and health situations, thereby reinforcing its significance in sustainable and precision livestock production.

## 5 Future research

Future research can build on this study in multiple ways. First, bioelectrical impedance analysis (BIA) can be used to diagnose more animal health issues. BIA can detect tissue changes caused by metabolic abnormalities, dietary deficits, and chronic illnesses. Studying BIA in these circumstances could yield useful insights and non-invasive ways for early diagnosis of livestock (including ruminants and small ruminants) health concerns. Further research into deep learning models may improve livestock examinations machine learning prediction. Test more advanced neural networks like CNNs or RNNs to determine whether they improve the present models. When paired with larger datasets or time-series data from repeated BIA tests, these models may better capture temporal trends and long-term dependencies. Researchers could construct algorithms that detect and forecast parasitism by tracking goats and collecting BIA data at different phases of infection. Early intervention and improved herd health and productivity may result from more effective treatment regimens. These methods could also be applied to sheep, and cattle to test the generalizability of BIA and machine learning models across farming systems. Understanding how parasitism or other health issues affect each species’ bioelectrical characteristics can help build species-specific diagnostic tools. Finally, future research should also examine the economic benefits of BIA and machine learning for health issues identification. Early infection prevention can save treatment costs, productivity losses, and animal welfare, improving farm profitability and sustainability.

## Data Availability

The raw data supporting the conclusions of this article will be made available by the authors, without undue reservation.

## References

[1] Arsenopoulos KV Fthenakis GC Katsarou EI Papadopoulos E. Haemonchosis: a challenging parasitic infection of sheep and goats. Animals. (2021) 11:363. doi: 10.3390/ani11020363, PMID: 33535656 PMC7912824

[2] Bunyan A. Investigations into the population structure of the ovine parasitic nematode Haemonchus Contortus: a comparison of isolates from differing climatic and geographical regions of New South Wales. Bathurst: Charles Sturt University (2021).

[3] Casey SJ. Haemonchus Contortus infections in alpacas and sheep. Virginia: Virginia Tech (2014).

[4] Clark DAS. Haemonchus Contortus and hookworms—Parallels in vaccine development. Scotland: University of Glasgow (2006).

[5] Teddleton H. G. (2024). "Effect of Haemonchus contortus excretory/secretory protein on differences in host neutrophil migration." Available online at: https://researchrepository.wvu.edu/etd/12417 (Accessed January 10, 2025).

[6] Glaji YA Mani AU Bukar MM Igbokwe IO. Reliability of the FAMACHA© chart for the evaluation of Anaemia in goats in and around Maiduguri. Sokoto J Vet Sci. (2014) 12:9–14. doi: 10.4314/sokjvs.v12i3.2

[7] Kaplan RM Burke JM Terrill TH Miller JE Getz WR Mobini S. Validation of the FAMACHA© eye color chart for detecting clinical Anemia in sheep and goats on farms in the southern United States. Vet Parasitol. (2004) 123:105–20. doi: 10.1016/j.vetpar.2004.06.005, PMID: 15265575

[8] Cain JL Slusarewicz P Rutledge MH McVey MR Wielgus KM Zynda HM. Diagnostic performance of McMaster, Wisconsin, and automated egg counting techniques for enumeration of equine Strongyle eggs in fecal samples. Vet Parasitol. (2020) 284:109199. doi: 10.1016/j.vetpar.2020.109199, PMID: 32801106

[9] Verocai GG Chaudhry UN Lejeune M. Diagnostic methods for detecting internal parasites of livestock. Vet Clin N Am Food Anim Pract. (2020) 36:125–43. doi: 10.1016/j.cvfa.2019.12.003, PMID: 32029179

[10] Levecke B Kaplan RM Thamsborg SM Torgerson PR Vercruysse J Dobson RJ. How to improve the standardization and the diagnostic performance of the fecal egg count reduction test? Vet Parasitol. (2018) 253:71–8. doi: 10.1016/j.vetpar.2018.02.004, PMID: 29605007

[11] Ljungström S Melville L Skuce PJ Höglund J. Comparison of four diagnostic methods for detection and relative quantification of Haemonchus contortus eggs in feces samples. Front Vet Sci. (2018) 4:239. doi: 10.3389/fvets.2017.00239, PMID: 29417052 PMC5787577

[12] Bosco A. The Coprological diagnosis of gastrointestinal nematode infections in small ruminants. Italy: Università degli Studi di Napoli Federico II (2014).

[13] Demelash K Abebaw M Negash A Alene B Zemene M Tilahun M. A review on diagnostic techniques in veterinary helminthology. Nat Sci. (2016) 14:109–18.

[14] Bentounsi B Attir B Meradi S Cabaret J. Repeated treatment Faecal egg counts to identify gastrointestinal nematode resistance in a context of low-level infection of sheep on farms in eastern Algeria. Vet Parasitol. (2007) 144:104–10. doi: 10.1016/j.vetpar.2006.09.013, PMID: 17067742

[15] Rinaldi L Veneziano V Morgoglione ME Pennacchio S Santaniello M Schioppi M. Is gastrointestinal Strongyle fecal egg count influenced by hour of sample collection and worm burden in goats? Vet Parasitol. (2009) 163:81–6. doi: 10.1016/j.vetpar.2009.03.043, PMID: 19414222

[16] Ngere L Burke JM Morgan JLM Miller JE Notter DR. Genetic parameters for fecal egg counts and their relationship with body weights in Katahdin lambs. J Anim Sci. (2018) 96:1590–9. doi: 10.1093/jas/sky064, PMID: 29635633 PMC6140914

[17] Kushner RF. Bioelectrical impedance analysis: a review of principles and applications. J Am Coll Nutr. (1992) 11:199–209. doi: 10.1080/07315724.1992.12098245 1578098

[18] Valentinuzzi ME Morucci JP Felice CJ. Bioelectrical impedance techniques in medicine part II: monitoring of physiological events by impedance. Crit Rev Biomed Eng. (1996) 24:353–466. doi: 10.1615/CritRevBiomedEng.v24.i4-6.30, PMID: 9196885

[19] Davydov DM Boev A Gorbunov S. Making the choice between bioelectrical impedance measures for body hydration status assessment. Sci Rep. (2021) 11:7685. doi: 10.1038/s41598-021-87253-4, PMID: 33833322 PMC8032770

[20] Gadir G Gunay K. Measurement of electrical conductivity of biologically active points. Endless Light Sci. (2023) 5:281–6.

[21] Jackson AA Johnson M Durkin K Wootton S. Body composition assessment in nutrition research: value of BIA technology. Eur J Clin Nutr. (2013) 67:S71–8. doi: 10.1038/ejcn.2012.167, PMID: 23299874

[22] Marra M Sammarco R De Lorenzo A Iellamo F Siervo M Pietrobelli A. Assessment of body composition in health and disease using bioelectrical impedance analysis (BIA) and dual-energy X-ray absorptiometry (DXA): a critical overview. Contrast Media Mol Imaging. (2019) 2019:1–9. doi: 10.1155/2019/3548284, PMID: 31275083 PMC6560329

[23] Vapnik V Golowich S Smola A. Support vector method for function approximation, regression estimation and signal processing In: Mozer M Jordan M Petsche T, editors. Advance in neural information processing system. Cambridge: MIT Press (1996)

[24] Weston J Mukherjee S Chapelle O Pontil M Poggio T Vapnik V. Feature selection for SVMs. Adv Neural Inf Proces Syst. (2000) 12:668–74.

[25] Winston PH. Artificial intelligence. Massachusetts: MIT Open Course Ware (2024).

[26] Cortes C Vapnik V. Support-vector networks. Mach Learn. (1995) 20:273–97. doi: 10.1007/BF00994018

[27] Hinton G. E. Srivastava N. Krizhevsky A. Sutskever I. Salakhutdinov R. R.. (2012). "Improving neural networks by preventing co-adaptation of feature detectors." Available online at: https://arxiv.org/abs/1207.0580 (Accessed January 15, 2025).

[28] Siddique A Herron CB Wu B Melendrez KS Sabillon LJ Garner LJ. Development of predictive classification models and extraction of signature wavelengths for the identification of spoilage in chicken breast fillets during storage using near infrared spectroscopy. Food Biopr Technol. (2024) 13:1–9. doi: 10.21203/rs.3.rs-4478852/v1

[29] Siddique A. (2023). Implementing big data analytics approaches to improve food quality and minimize food waste and loss. Available online at: https://etd.auburn.edu//handle/10415/8709 (Accessed February 10, 2025).

[30] Siddique A Shirzaei S Smith AE Valenta J Garner LJ Morey A. Acceptability of artificial intelligence in poultry processing and classification efficiencies of different classification models in the categorisation of breast fillet myopathies. Front Physiol. (2021) 12:712649. doi: 10.3389/fphys.2021.712649, PMID: 34630138 PMC8493215

[31] Halder RK Uddin MN Uddin MA Aryal S Khraisat A. Enhancing K-nearest neighbor algorithm: a comprehensive review and performance analysis of modifications. J Big Data. (2024) 11:113. doi: 10.1186/s40537-024-00973-y

[32] Siddique A Herron CB Valenta J Garner LJ Gupta A Sawyer JT. Classification and feature extraction using supervised and unsupervised machine learning approach for broiler Woody breast myopathy detection. Food Secur. (2022) 11:3270. doi: 10.3390/foods11203270, PMID: 37431018 PMC9601423

[33] Altman NS. An introduction to kernel and nearest-neighbor nonparametric regression. Am Stat. (1992) 46:175–85. doi: 10.1080/00031305.1992.10475879

[34] Pratap B Bansal S. Optimizing artificial neural network using genetic algorithm In: Sumit S, editor. Bio-Inspired Optimization for Medical Data Mining. New York: Wiley (2024). 269–88.

[35] Vasconcelos GA Francisco MB da Costa LR Ribeiro Junior RF Melo MD. Prediction of surface roughness in duplex stainless steel face milling using artificial neural network. J Adv Manuf Technol. (2024) 133:2031–8. doi: 10.1007/s00170-024-13955-4

[36] Padhy R Dash SK Khandual A Mishra J. Image classification in artificial neural network using fractal dimension. Int J Inf Technol. (2023) 15:3003–13. doi: 10.1007/s41870-023-01318-3

[37] Chen T. Guestrin C.. (2016). "XGBoost: a scalable tree boosting system." In Proceedings of the 22nd ACM SIGKDD international conference on knowledge discovery and data mining, 785–794. Association for Computing Machinery. New York, NY.

[38] Sarker IH. Deep learning: a comprehensive overview on techniques, taxonomy, applications and research directions. SN Comput Sci. (2021) 2:420. doi: 10.1007/s42979-021-00815-1, PMID: 34426802 PMC8372231

[39] Brownlee J. Develop deep learning models on Theano and tensor flow using Keras. J Chem Inf Model. (2019) 53:1689–99.

[40] Taye MM. Understanding of machine learning with deep learning: architectures, workflow, applications and future directions. Computers. (2023) 12:91. doi: 10.3390/computers12050091

[41] Meadi M. N. Benbrahim H.. (2024). "Cervical spine fracture detection using deep learning algorithm." In Proceedings of the 8th international conference on image and signal processing and their applications (ISPA), 1–8. IEEE: New Jersey.

[42] Ehsan M Hu RS Liang QL Hou JL Song X Yan R. Advances in the development of anti-Haemonchus contortus vaccines: challenges, opportunities, and perspectives. Vaccine. (2020) 8:555. doi: 10.3390/vaccines8030555, PMID: 32971770 PMC7565421

[43] Mares MM Abdel-Gaber R Murshed M Aljawdah H Al-Quraishy S. In vitro anthelmintic activity of *Croton tiglium* seeds extract on Haemonchus contortus. Indian J Anim Res. (2023) 57:1703–6. doi: 10.18805/IJAR.BF-1670

[44] Soliman SM Salem HM El-Saadony M Ahmed AE Saad A El-Saadony MT. Haemonchus contortus infection of goats and the use of anthelmintic natural alternative: an updated review. J Hellenic Vet Med Soc. (2024) 75:7201–10. doi: 10.12681/jhvms.29905

[45] Buchmann K Christiansen LL Kania PW Thamsborg SM. Introduced European bison (*Bison bonasus*) in a confined forest district: a ten year parasitological survey. Int J Parasitol Parasites Wildl. (2022) 18:292–9. doi: 10.1016/j.ijppaw.2022.07.005, PMID: 35934997 PMC9350870

[46] Zajac AM Conboy GA Greiner EC Smith SA Snowden KF. Fecal examination for the diagnosis of parasitism. Vet Clini Parasit. (2012) 8:72–3.

[47] Cao Y He Y Bai H. Feature scaling optimization in machine learning. IEEE Access. (2020) 8:112154–65.

[48] Roy S Mukherjee A Biswas A. A comprehensive study of scaling in machine learning models. Int J Modern Trends Sci Technol. (2021) 7:11–7.

[49] Zhang S Yao L Sun A Tay Y. Deep learning-based recommender system: a survey and new perspectives. ACM Comput Surv. (2019) 52:1–38.

[50] Hastie T Tibshirani R Friedman JH. The elements of statistical learning. New York: Springer (2009).

[51] Cawley GC Talbot NL. On over-fitting in model selection and subsequent selection Bias in performance evaluation. J Mach Learn Res. (2010) 11:2079–107.

[52] Varoquaux G Raamana PR Engemann DA Hoyos-Idrobo A Schwartz Y Thirion B. Assessing and tuning brain decoders: cross-validation, caveats, and guidelines. Neuroimage. (2017) 145:166–79. doi: 10.1016/j.neuroimage.2016.10.038, PMID: 27989847

[53] Bishop CM. Neural networks for pattern recognition. Oxford: Oxford University Press (1995).

[54] Pedregosa F Varoquaux G Gramfort G Michel V Thirion B Grisel O. Scikit-learn: machine learning in Python. J Mach Learn Res. (2011) 12:2825–30.

[55] Jordan MI Mitchell TM. Machine learning: trends, perspectives, and prospects. Science. (2015) 349:255–60. doi: 10.1126/science.aaa8415, PMID: 26185243

[56] Bradley AP. The use of the area under the ROC curve in the evaluation of machine learning algorithms. Pattern Recogn. (1997) 30:1145–59. doi: 10.1016/S0031-3203(96)00142-2

[57] Kumar D Das S Kumar D. Ensemble learning techniques: an overview. Int J Mach Learn Networked Collab Eng. (2021) 1:1–12.

[58] McKinney W. (2010). "Data structures for statistical computing in Python." In Proceedings of the 9th Python science conference, 445: 51–56. Austin: SciPy

[59] Friedman JH. Greedy function approximation: a gradient boosting machine. Ann Stat. (2001) 29:1189–232. doi: 10.1214/aos/1013203451, PMID: 38281721

[60] Goodfellow I Bengio Y Courville A. Deep learning. Cambridge, MA: MIT Press (2016).

[61] Seber GAF Lee AJ. Linear regression analysis. Hoboken, NJ: John Wiley & Sons (2012).

[62] Shome A Mukherjee G Chatterjee A Tudu B. Study of different regression methods, models and application in deep learning paradigm In: Mukherjee G, editor. Deep learning concepts in operations research. Boca Raton, FL: Auerbach Publications (2024). 130–52.

[63] Varma S Simon R. Bias in error estimation when using cross-validation for model selection. BMC Bioinformatics. (2006) 7:1–8. doi: 10.1186/1471-2105-7-91, PMID: 16504092 PMC1397873

[64] Kuhn M Johnson K. Applied predictive modeling. New York: Springer (2013).

[65] Aburto-Corona JA Calleja-Núñez JJ Moncada-Jiménez J de Paz JA. The effect of passive dehydration on phase angle and body composition: a bioelectrical impedance analysis. Nutrients. (2024) 16:2202. doi: 10.3390/nu16142202, PMID: 39064645 PMC11279509

[66] Hioka A Akazawa N Okawa N Nagahiro S. Extracellular water-to-Total body water ratio is an essential confounding factor in bioelectrical impedance analysis for sarcopenia diagnosis in women. Eur Geriatr Med. (2022) 13:789–94. doi: 10.1007/s41999-022-00652-2, PMID: 35536459

[67] Hoste H Torres-Acosta JFJ Quijada J Chan-Perez I Dakheel MM Kommuru DS. Interactions between nutrition and infections with Haemonchus contortus and related gastrointestinal nematodes in small ruminants. Adv Parasitol. (2016) 93:239–51. doi: 10.1016/bs.apar.2016.02.025, PMID: 27238007

[68] Brito DRB Júnior LMC Garcia JL Chaves DP Júnior JAAC Conceição WLF. Clinical parameters of goats infected with gastrointestinal nematodes and treated with condensed tannin. Semina. (2020) 41:517–30. doi: 10.5433/1679-0359.2020v41n2p517

[69] Moro AB Galvani DB Montanholi YR Bertemes-Filho P Venturini RS Martins AA. Assessing the composition of the soft tissue in lamb carcasses with bioimpedance and accessory measures. Meat Sci. (2020) 169:108192. doi: 10.1016/j.meatsci.2020.108192, PMID: 32485563

[70] Shim G Breinyn IB Martínez-Calvo A Rao S Cohen DJ. Bioelectric stimulation controls tissue shape and size. Nat Commun. (2024) 15:2938. doi: 10.1038/s41467-024-47079-w, PMID: 38580690 PMC10997591

[71] Ward LC Brantlov S. Bioimpedance basics and phase angle fundamentals. Rev Endocr Metab Disord. (2023) 24:381–91. doi: 10.1007/s11154-022-09780-3, PMID: 36749540 PMC10140124

[72] Gulli A Kapoor A Pal S. Deep learning with tensor flow 2 and Keras: Regression, conv nets, GANs, RNNs, NLP, and more with the Keras API. Birmingham, UK: Packt Publishing Ltd (2019).

[73] Hecht-Nielsen R. (1988). "Theory of the backpropagation neural network." In Proceedings of the international joint conference on neural networks, 593–605. University of California, San Diego.

[74] LeCun Y Bengio Y Hinton G. Deep learning. Nature. (2015) 521:436–44. doi: 10.1038/nature14539, PMID: 26017442

[75] Scholkopf B Smola AJ. Learning with kernels: Support vector machines, regularization, optimization, and beyond. Cambridge, MA: MIT Press (2002).

[76] Caruana R. Niculescu-Mizil A.. (2006). "An empirical comparison of supervised learning algorithms." In Proceedings of the 23rd international conference on machine learning (ICML), 161–168. Association for Computing Machinery. New York, NY.

[77] Rumelhart DE Hinton GE Williams RJ. Learning representations by Back-propagating errors. Nature. (1986) 323:533–6. doi: 10.1038/323533a0

[78] Smola AJ Schölkopf B. A tutorial on support vector regression. Stat Comput. (2004) 14:199–222. doi: 10.1023/B:STCO.0000035301.49549.88

[79] Moro C Stromberga Z Moreland A. Enhancing teaching in biomedical, health and exercise science with real-time physiological visualisations. Adv Exp Med Biol. (2020) 1260:1–11. doi: 10.1007/978-3-030-47483-6_1 33211304

[80] Utkarsh A Jain PK. Predicting bentonite swelling pressure: optimized XGBoost versus neural networks. Sci Rep. (2024) 14:17533. doi: 10.1038/s41598-024-68038-x, PMID: 39080334 PMC11289295

[81] Berg EP Marchello MJ. Bioelectrical impedance analysis for the prediction of fat-free mass in lambs and lamb carcasses. Journal of Animal Science. (1994) 72: 322–329. 8157516 10.2527/1994.722322x

[82] Wuenschel MJ McElroy WD Oliveira K Richard S McBride RS. Measuring fish condition: an evaluation of new and old metrics for three species 2 contrasting life histories. Can J Fish Aquat Sci. (2018) 1–64.

[83] Saiz del Barrio A García-Ruiz AI Fuentes-Pila J Nicodemus N. Application of bioelectrical impedance analysis (BIA) to assess carcass composition and nutrient retention in rabbits from 25 to 77 days of age. Animals. (2022) 12:2926. 36359050 10.3390/ani12212926 PMC9654842

[84] Rae LS Rand JS Ward LC. Measuring body composition in dogs using bioelectrical impedance spectroscopy. The Veterinary Journal. (2024) 304:106067. 38266810 10.1016/j.tvjl.2024.106067

[85] Arsenopoulos KV Minoudi S Symeonidou I Triantafyllidis A Fthenakis GC Papadopoulos E. Extensive countrywide molecular identification and high genetic diversity of Haemonchus spp. in domestic ruminants in Greece. Pathogens. (2024) 13:238. 38535581 10.3390/pathogens13030238 PMC10974071

